# Safety, Efficiency, and Outcomes of Perineoplasty: Treatment of the Sensation of a Wide Vagina

**DOI:** 10.1155/2016/2495105

**Published:** 2016-08-17

**Authors:** Mustafa Ulubay, Ugur Keskin, Ulas Fidan, Mustafa Ozturk, Serkan Bodur, Ali Yılmaz, Mehmet Ferdi Kinci, Mufit Cemal Yenen

**Affiliations:** ^1^Obstetrics and Gynecology Department, Gulhane Military Medical Academy (GATA), Etlik, 06018 Ankara, Turkey; ^2^Obstetrics and Gynecology Department, Etimesgut Military Hospital, Etimesgut, 06100 Ankara, Turkey; ^3^Obstetrics and Gynecology Department, Haydarpasa Military Training Hospital, Gulhane Military Medical Academy (GATA), 34668 Istanbul, Turkey

## Abstract

*Background*. The sensation of a wide vagina is a common problem for women after childbirth. As its etiology is unknown, there is no uniform management strategy. We hypothesized that, rather than vaginal laxity, the cause was level 3 pelvic support deficiency.* Methods*. This retrospective study compared preoperative and postoperative genital hiatus length, perineal length, and total vaginal length in patients treated with perineoplasty for the sensation of a wide vagina. A telephone survey was used to determine postoperative patient and male partner satisfaction rates.* Results*. Mean age of patients was 48 (26–68) years; mean body mass index (BMI) was 25.3 (17.6–33.2); and mean parity was 2.5 (2–5). Preoperative and postoperative genital hiatus, perineal length, and total vaginal length were 4.62 and 3.18 (*p* < 0.01), 3.06 and 4.04 (*p* < 0.01), and 9.43 and 9.43 (*p* = 0.882), respectively. At the 6-month follow-up, the success rate of the perineoplasty procedure was 87.9%; according to a visual analog scale, partner satisfaction rate was 92.6%. Ten percent (*n* = 4) of patients said they experienced dyspareunia during sexual intercourse at the introitus of the vagina.* Conclusion*. With low dyspareunia rates, low complication rates, high patient satisfaction, and satisfactory anatomical success, perineoplasty can be considered successful for treatment of the sensation of a wide vagina.

## 1. Introduction 

Following vaginal labor, some women complain of the sensation of a wide vagina because of severe perineal lacerations, incorrectly repaired episiotomy techniques, or diminished pelvic support [1], but there are few data on this phenomenon in the literature. Depending on age, sexual dysfunction—including decreased libido, vaginal dryness, inability to achieve orgasm, and dyspareunia—is common in up to 40% of women [2]. Women with the sensation of a wide vagina may complain of decreased friction during coitus and diminished sexual satisfaction, and their male partners may also complain about reduced friction during coitus. Although several nonsurgical and surgical techniques and modifications have been described for the treatment of the sensation of a wide vagina, there is no uniform approach to management [3, 4].

For such patients, vaginoplasty to narrow the vaginal caliber is one option, but surgeons have long favored anterior colporrhaphy, posterior colporrhaphy, lateral colporrhaphy, perineoplasty, colpoperineoplasty, or some combination of these [3–5]. Surgeons routinely perform colpoperineoplasty or vaginoplasty as a “one fits all” solution to wide vagina sensation, performing vaginal mucosal excision procedures to treat these patients by narrowing the vaginal caliber [6, 7].

Perineoplasty may be performed in a single operation or as one of a series of concomitant surgical procedures in patients with rectal or pelvic organ prolapse [8]. In particular, it is performed to maintain level 3 pelvic support in cases of pelvic organ prolapse, as explained by DeLancey [9]. The procedure may also be performed for cosmetic reasons at the patient's request to excise perineal scatrises or for vulvar vestibulitis and vulvar lichen sclerosis treatment with stenotic vaginal introitus [3, 6, 10]. Perineoplasty may also be performed to improve sexual satisfaction and decrease dyspareunia caused by incorrect episiotomy repair [11].

Although perineoplasties have been performed many times and are associated with very low complication rates, there are no established or uniform surgical methods for performing the procedure [12]. Additionally, there are no published guidelines on patient selection criteria for perineoplasty [12]. In our practice, we perform perineoplasty rather than vaginal mucosal excision to treat women with the sensation of a wide vagina, on the assumption that the cause is impaired pelvic support rather than vaginal mucosal laxity. The aim of the present study was to describe the use of perineoplasty for the treatment of this condition and to investigate its efficiency, safety, and outcomes.

## 2. Material and Methods 

In this retrospective study, which received local ethical approval, we reviewed the patient records of women who had presented with sensation of a wide vagina between January 2012 and September 2015, who had requested surgical treatment and were treated with perineoplasty. Sixty-four patients were initially enrolled in the study; all were told that participation in the study was voluntary, and all provided verbal informed consent. Inclusion criteria were that patients had had at least one vaginal delivery, requested surgery for wide vagina sensation, underwent perineoplasty, and had regular or irregular sexual intercourse following the operation. A telephone survey was performed to identify patients who wished to participate in the study. Of these, 12 patients did not meet the inclusion criteria, 8 patients declined to participate, and 6 patients could not be reached.

All the patients underwent a gynecological examination in the lithotomy position, and the pelvic organ prolapse quantification (POP-Q) scoring system was applied in all cases [13]. The gynecologist did not suggest the need for perineoplasty following a pelvic examination for the sensation of a wide vagina, with the exception of one case in which a patient requested treatment. In that instance, the gynecologist provided information about relevant surgical techniques, including perineoplasty. Patients with pelvic organ prolapse greater than POP-Q stage ≥ 1, rectocele, cystocele, previous perineoplasty or vaginoplasty operations, dyspareunia, anal incontinence, obstructed defecation, sexually transmitted diseases, and cervical smear abnormalities and patients who were pregnant or had delivered within the previous 12 months were excluded from the study.

All patients underwent a subsequent examination one week after surgery to record early postoperative complications. POP-Q scoring was again performed 6 months after surgery to detect late complications. As there are no validated quality of life tests for the sensation of a wide vagina, a nonvalidated test was prepared in the form of a telephone survey. At least 6 months after surgery, all the study participants (*n* = 38) were contacted by telephone and asked to take part in the survey as follows.

*Telephone Survey Questions (Following Surgery)*

1. Please rate the success of the operation in narrowing your vagina. (0–100 points)
2. Please rate male partner satisfaction as regards narrowing of the vaginal caliber. (0–100 points)
3. Please state whether you would recommend the operation to a friend.
4. Did you experience any dyspareunia following surgery?
  - Yes (please state when)
  - No.

### 2.1. Surgical Procedure

General or local anesthesia was performed at the request of either the patient or the anaesthesiologist. Intraoperatively, 2 g of cefazolin was administered intravenously as an antibiotic prophylaxis to all patients other than those with allergies. Each patient was placed in the lithotomy position; 10% povidone-iodine was applied to the skin, and the patient was draped. The posterior vaginal fourchette was elevated with Kocher clamps bilaterally at the border of the hymen, and a transverse incision was made using a number 11 scalpel. Another clamp was used to elevate the perineal skin at a distal 1/3 length of the perineal length near the anus. The clamps were used to hold the perineal skin, and a vertical incision was made, using a number 11 scalpel, from the centrum of the posterior fourchette to the anus until the distal 1/3 length of the perineal area was reached. The perineal skin was dissected with scissors or a scalpel, and remnant tissue was trimmed bilaterally. The perineal body was strengthened, and the superficial transverse perineal muscle was approximated with transverse interrupted sutures in the midline of the two layers. The rectovaginal fascia was approximated to the perineal body using interrupted sutures. The skin was also approximated with interrupted sutures. Bulbocavernosus muscle was approximated with sutures at the level of the posterior fourchette.

All patients were discharged on postoperative day 1. No postoperative complications occurred intraoperatively.

### 2.2. Statistical Methods

SPSS version 15.0 (Chicago, IL, USA) was used for all statistical analyses. Data are expressed as mean ± standard deviation. Paired *t*-testing was used for comparison of variables.

## 3. Results

The mean age of participating patients was 48 (range 26–68). The mean body mass index (BMI) was 25.3 (17.6–33.2), and mean parity was 2.5 (2–5). Mean preoperative and postoperative genital hiatus, perineal length, and total vaginal length were 4.62 and 3.18 (*p* < 0.01), 3.06 and 4.04 (*p* < 0.01), and 9.43 and 9.43 (*p* = 0.882), respectively. Anatomical preoperative and postoperative measurements are shown in Table 1.

In response to the first survey question (“Please rate the success (0–100 points) of the operation in narrowing the vagina”), patients' reported satisfaction rate was 87.9%  ± 6.85. For the second question (“Please rate the satisfaction of your husband (0–100 points) as regards the narrowing of the vaginal caliber”), reported male partner satisfaction rate was 92.63%  ± 7.6. In response to the third question (“Please state whether you would recommend this operation to your friends”), 87.9%  ± 6.8 of patients said they would recommend the procedure to a friend. For the fourth question (“Did you experience any dyspareunia after the surgery?”), 10% (*n* = 4) of patients said they experienced dyspareunia at the introitus of the vagina during sexual intercourse.

There were no intraoperative complications. Two cases of early suture separation occurred in the first week after the surgery, but these did not require resuturation. Characteristics of the patients are set out in Tables 2 and 3.

Concomitantly with the perineoplasty, 10 of the patients underwent transobturator tape operations to treat stress urinary incontinence; one patient underwent laparoscopic tubal sterilization upon request; and five underwent diagnostic hysteroscopy for abnormal uterine bleeding. Two patients had a total abdominal hysterectomy; one had a vaginal hysterectomy for abnormal uterine bleeding; and four had a mastectomy due to breast cancer. Two patients had an appendectomy in their medical history.

## 4. Discussion

Based on anatomical descriptions, we proposed an explanation for the sensation of a wide vagina by women and their male partners as disruption of the structural integrity of the rectovaginal fascia and perineal body [14]. On this view, the pelvic support defect would be level 3, as described by DeLancey [9]. The principal aim of the perineoplasty was to restore the integrity of the rectovaginal fascia and perineal body. In the present study, rather than vaginal mucosal excision procedures involving anterior, posterior, or lateral approaches, the perineal body was fortified, and the superficial transverse perineal muscle was approximated in the midline, restoring the integrity of the rectovaginal fascia and perineal body and the circular structure of both bulbocavernosus muscles. Following perineoplasty, the patient satisfaction rate was 87.9%, and the satisfaction rate (positive sensation) of their male partners was 92.6%. The number of patients who said they would recommend the procedure to friends was also very high (87.9%).

In the present study, 10 (26.3%) of the patients underwent transobturator taping procedures concomitantly with the perineoplasty because of stress urinary incontinence. As a positive correlation has been reported between stress urinary incontinence and sexual dysfunction [15], it seems possible that pelvic support deficiency (as outlined above) may be responsible for the sensation of a wide vagina. In cases of stress urinary incontinence in women, we propose that the gynecologist or urogynecologist should inquire about the presence of that sensation. In the present study, 16 of 38 patients (42%) had concomitant surgery, suggesting that benign gynecological comorbidities other than sensation of a wide vagina may be present in many cases. Concomitant surgery should be performed for these comorbidities and sensation of a wide vagina.

A recent magnetic resonance imaging study reported the presence of tears in components of the levator ani muscles following vaginal delivery, in contrast to nulliparous women [16]. The same study suggested that pelvic floor and pubic bone attachment abnormalities were present in the vaginal delivery group. In vaginal delivery, levator ani muscle tears and perineal lacerations can lead to the sensation of a wide vagina. However, as most women attribute this phenomenon to the aging process, pelvic floor dysfunction caused by diminished pelvic floor support is probably overlooked.

There are few existing studies of the sensation of a wide vagina. Pardo et al. performed a combination of site-specific anterior and posterior repair, paravaginal suturing, and perineoplasty for such patients [4], reporting a patient satisfaction rate (as assessed by the sensation of a tighter vagina) of 96% at 6 months after the procedure. Patients in that study also reported improvements in sexual satisfaction following surgery [4]. An Iranian study of a combination of vaginal mucosal excision and perineoplasty for women with vaginal laxity reported a satisfaction rate of 97.5% after surgery [5]. However, the prevalence of dyspareunia increased, and vaginal lubrication decreased significantly. Weber et al. reported that while transvaginal colpoperineorrhaphy provided good anatomical support, 27% of patients experienced de novo dyspareunia [17, 18]. Although in the present study we did not perform vaginal mucosal excision procedures such as posterior or anterior colporrhaphy, 4 patients (10%) reported de novo dyspareunia following perineoplasty; some of the women reported dyspareunia at the introitus of the vagina during sexual intercourse.

Previous studies have suggested that scar tissue in the vagina, especially after colporrhaphy posterior operations, may be responsible for dyspareunia [3, 5]. Others have proposed that lateral colporrhaphy should be performed to reduce dyspareunia and to treat sensation of a wide vagina [3]. However, in all the previous studies, vaginal mucosal procedures were performed, which decrease vaginal mucosal volume. Although vaginal mucosal excision may appear a feasible and easy means of narrowing the vaginal caliber, the anatomical description of perineal support described above gives rise to some concerns. We believe that, in vaginal mucosal excision procedures, fibrosis and scar tissue lead to decreased blood flow to the vagina and fibrosis, resulting in vaginal dryness and dyspareunia [19]. As an alternative approach, we have sought to support the integrity of the rectovaginal fascia and perineal body, fortifying the perineal body and approximating the superficial transverse perineal muscle. Based on our findings and those in the literature, this approach seems successful in alleviating the sensation of a wide vagina and reducing the occurrence of de novo dyspareunia.

Although high short-term satisfaction rates have been reported for patients who have undergone anterior and posterior colporrhaphy and vaginoplasty or a combination of these procedures for the sensation of a wide vagina, data on long-term outcomes are limited [3–5]. This makes it difficult to determine whether mucosal excision procedures improve the sexual well-being of patients and their male partners [5]. According to one study, the rate of new dyspareunia following posterior colporrhaphy was 15%, with or without levator plication [20]. In the postmenopausal period, decreased vaginal laxity caused by reduced estrogenic activity and vaginal mucosal volume may account for dyspareunia. The literature provides no data on the long-term outcomes of patients treated with mucosal excision procedures. Based on the findings of the present study, we suggest that the anatomical approach described here may be preferable to mucosal excision.

In our clinical practice, we frequently perform anterior and posterior colporrhaphy and endopelvic fascia plication procedures for patients with rectocele and cystocele. However, it is difficult to create an optimum vaginal caliber for the patient that will not cause de novo dyspareunia because of a narrow vagina. Subjectively, the two-finger measurement method is not an objective means of assessing the optimum diameter of the vaginal caliber. Mellgren et al. reported prospective results of rectocele repair [18]. Of 53 patients who had posterior colporrhaphy, 9 (17%) complained of introital tightness following posterior colporrhaphy, suggesting that surgeons should be cautious about reducing vaginal caliber in this way.

In the current study, we selected perineoplasty rather than vaginal mucosa excision as a means of narrowing vaginal caliber because of the anatomical support it provides. After surgery, the dynamic structure of the perineal and posterior vaginal wall was modified, and strengthening of the perineal body provided extra support for the posterior vaginal wall during coitus. A postsurgery satisfaction rate of 87% indicates that perineoplasty alone may be sufficient to treat the sensation of a wide vagina, with reduced rates of de novo dyspareunia. Additionally, the nature of the perineoplasty procedure means that excessive iatrogenic narrowing of the vaginal caliber is not a problem.

Our experience suggests that when dissecting the skin of the perineum during perineoplasty, microsurgical scissors should be used. These can readily dissect the skin from underlying subcutaneous tissue without damage to the perineal musculature and provide an intense dissecting cleavage plane with little bleeding. In pelvic reconstructive surgery, it is important to use such scissors and equipment designed for surgical dissections. In the present study, we did not perform levatororaphy in any of the patients because of its reported effects on dyspareunia [21].

When preoperative and postoperative perineal lengths were compared, our findings show that perineal length measurements increased by 30%. In addition, genital hiatus measurements decreased by 40% postoperatively. There was no statistically significant decrease between preoperative and postoperative measurements of total vaginal length. We suggest that the increase in perineal length and fortification of the perineal body may be one reason for the sensation of narrowing of the vagina after perineoplasty reported by women and their male partners. As described earlier, restoring the integrity of the rectovaginal fascia and supporting the perineal body provide level 3 pelvic support [9, 14].

The present study has some limitations. It is purely descriptive, and the small sample size means that randomization could not be performed. In addition, the lack of available data on the sensation of a wide vagina meant that it was not possible to use validated quality of life questionnaires. The study included only women who had regular or intermittent sexual intercourse following surgery, which does not reflect the real population of those experiencing vaginal laxity; it may be that those who are not having a sex life may have dyspareunia.

Prospective randomized controlled trials are needed to assess the effectiveness of perineoplasty as compared to colpoperineoplasty in patients with the sensation of a wide vagina. The potential of nonsurgical treatments such as physiotherapy or laser therapy should also be evaluated, and there is a need to develop validated quality of life questionnaires.

## 5. Conclusions

Perineoplasty to address the sensation of a wide vagina seems to be associated with high patient satisfaction rates and high male partner satisfaction rates, with low complication and de novo dyspareunia rates in the short term. While future studies should assess long-term outcomes, the present findings suggest that perineoplasty may offer a safe, efficient, and easy method of alleviating the sensation of a wide vagina.

## Figures and Tables

**Table 1:** Anatomical measurements before and after perineoplasty.

	*N*	Mean (mm)	Std. deviation	*p* value
Preoperative genital hiatus	38	4.62	0.93	<0.01
Postoperative genital hiatus	38	3.18	0.60	

Preoperative perineal length	38	3.06	0.49	<0.01
Postoperative perineal length	38	4.04	0.46	

Preoperative total vaginal length	38	9.43	1.31	0.882
Postoperative total vaginal length	38	9.43	1.29	

**Table 2:** Characteristics of participants.

	*N*	Minimum	Maximum	Mean	Std. deviation
Age	38	26	68	46.00	10.369
Parity	38	2	5	2.50	.762
BMI	38	17.6	33.2	25.3	.030
Question 1	38	75	100	87.79	6.815
Question 2	38	70	100	92.63	7.600
Question 3	38	75	100	87.92	6.808

**Table 3:** Dyspareunia rates.

	*N*	Yes	No
Question 4	38	4 (10%)	34 (90%)
